# Relative efficiency of joint-model and full-conditional-specification multiple imputation when conditional models are compatible: The general location model

**DOI:** 10.1177/0962280216665872

**Published:** 2016-09-05

**Authors:** Shaun R Seaman, Rachael A Hughes

**Affiliations:** 1 MRC Biostatistics Unit, Institute of Public Health, Cambridge, UK; 2 School of Social and Community Medicine, University of Bristol, Bristol, UK

**Keywords:** Compatibility, chained equations, congeniality, Gibbs sampler, informative margins, linear discriminant analysis, log linear model, missing data

## Abstract

Estimating the parameters of a regression model of interest is complicated by missing data on the variables in that model. Multiple imputation is commonly used to handle these missing data. Joint model multiple imputation and full-conditional specification multiple imputation are known to yield imputed data with the same asymptotic distribution when the conditional models of full-conditional specification are compatible with that joint model. We show that this asymptotic equivalence of imputation distributions does not imply that joint model multiple imputation and full-conditional specification multiple imputation will also yield asymptotically equally efficient inference about the parameters of the model of interest, nor that they will be equally robust to misspecification of the joint model. When the conditional models used by full-conditional specification multiple imputation are linear, logistic and multinomial regressions, these are compatible with a restricted general location joint model. We show that multiple imputation using the restricted general location joint model can be substantially more asymptotically efficient than full-conditional specification multiple imputation, but this typically requires very strong associations between variables. When associations are weaker, the efficiency gain is small. Moreover, full-conditional specification multiple imputation is shown to be potentially much more robust than joint model multiple imputation using the restricted general location model to mispecification of that model when there is substantial missingness in the outcome variable.

## 1 Introduction

Estimating the parameters of a regression model of interest (the ‘analysis model’) is often complicated in practice by missing data on the variables in that model. Multiple imputation (MI) is a popular method for dealing with this problem.^1^ Values for the missing variables are randomly sampled conditional on the observed variables from distributions thought approximately to describe the association between these variables. The result is an imputed dataset, in which there are no missing data. This imputation is done multiple (say, *M*) times and the analysis model is fitted separately to each of the resulting *M* imputed datasets to produce *M* estimates of the parameters β of this model. Finally, these *M* estimates are averaged to give an overall estimate of β, known as the ‘Rubin's Rules (point) estimate’.

MI methods differ in how they randomly sample values for the missing variables. The two most commonly used methods are joint model MI and full-conditional specification (FCS) MI (also known as MI by chained equations).^2,3^ The former involves specifying a joint model for the partially observed variables given the fully observed variables and sampling missing values from their posterior predictive distribution given the observed data. The latter involves specifying a conditional model for each of the partially observed variables given all the other variables and cycling through these models. In special cases, the two approaches are equivalent.^4^ For example, when all the conditional models in FCS MI are linear regressions with main effects and no interactions, FCS MI corresponds to joint model MI using a multivariate normal joint model. Likewise, when all the variables are categorical and the conditional models are saturated logistic regressions, FCS MI is equivalent to joint model MI using a saturated log linear joint model. In general, however, FCS MI is not equivalent to joint model MI.

Liu et al.^5^ (see also Zhu and Raghunathan^6^) showed that, even when FCS MI does not correspond to joint model MI, the distributions from which the two methods sample the missing values (the ‘imputation distributions’) are asymptotically the same when the conditional models used by FCS MI are compatible with a joint model. Compatibility is defined in Section 2. Although this is an important result, the ultimate purpose of MI is to enable the estimation of β, and it is unclear what the consequence of asymptotic equivalence of imputation distributions is for the relative efficiency (RE) of the Rubin's Rules estimator from FCS MI compared to that from joint model MI. This RE (i.e. the ratio of repeating-sampling variances of the two estimators of β) and, in particular, the asymptotic RE (the ratio as the sample size and *M* tend to infinity) is the focus of the current article.

When, as is commonly the situation, the partially observed variables consist of both continuous and categorical variables, the conditional models usually employed for them in FCS MI are linear regressions and multinomial logistic regressions, respectively. These are natural choices and are the default options in many statistical packages, e.g. *mice* and *mi* in R, and *ice* and *mi* in STATA. It can be shown that this set of conditional models is compatible with a restricted general location (RGL) joint model. Thus, Liu et al.'s (2014) result implies that FCS MI and joint model MI using the RGL model produce imputations that are asymptotically from the same distribution. Schafer^7^ described how to carry out joint model MI using this RGL model and provided software. So, joint model MI using the RGL model and FCS MI using conditional models compatible with this model are both options for the practicing statistician.

In the current article, we focus on the situation where the aim is to estimate the parameters of the analysis model and MI is used to handle missing data in the variables of that model. We elucidate the relation between joint model MI and FCS MI using compatible conditional models. We focus on the important case where joint model MI uses the RGL model and the (compatible) analysis model is a linear or logistic regression with parameters β. Our goals are (i) to demonstrate that when the RGL model is correctly specified, asymptotic equivalence of imputation distributions does not imply equally asymptotically efficient estimators of β; (ii) to investigate the magnitude of this difference and how it depends on the strength of associations between outcome and covariates in the analysis model and (iii) to demonstrate that when the joint distribution of the covariates implied by the RGL model is misspecified, FCS MI can be less biased than joint model MI. These goals will be realised using asymptotic calculations and simulation studies.

The structure of the article is as follows. In Section 2, we describe FCS and joint model MI in general and discuss how they are related when the conditional models are compatible. This relation can be one of equivalence in finite samples or asymptotic equivalence. The RGL model is introduced in Section 3. In Section 4, the asymptotic RE of inference from FCS MI with compatible conditional models versus that from the corresponding joint model MI is explored in depth for simple cases of the RGL model: one with a binary and two continuous variables, and one with four binary variables. In addition, the RE of the two MI methods is explored in a more complex situation using data simulated from a realistic data-generating mechanism based on the Barry Caerphilly Growth Study (BCGS).^8^ In Section 5, we discuss and illustrate, using simulated data and data from the National Childhood Development Study (NCDS),^9^ the relative robustness of FCS MI and joint model MI to misspecification of the joint model for the covariates implied by the RGL model. Section 6 contains a discussion.

## 2 Relation between FCS MI and joint model MI

Let $X=(X_{1},\ldots ,X_{K}{)}^{⊤}$ denote a vector of *K* variables, let $X_{-k}=(X_{1},\ldots ,$ $X_{k-1},$ $X_{k+1},\ldots ,X_{K}{)}^{⊤}$ and let *R*_*k*_ = 1 if *X*_*k*_ is observed and *R*_*k*_ = 0 if *X*_*k*_ is missing. We use subscript *i* to index the individual in the dataset (i=1,…,n). So, $X_{i}=(X_{i1},\ldots ,X_{\mathrm{iK}}{)}^{⊤},X_{i,-k}$ and *R*_*ik*_ denote the values of $X,X_{-k}$ and *R*_*k*_ for individual *i*. Let $M_{k}$ denote the set of indices of the individuals for whom *R*_*ik*_ = 0.

In joint model MI, a model f(X|θ) is specified for the joint distribution of X, with a non-informative prior p(θ) for the parameters θ in this model. Let Θ denote the parameter space of θ and assume that p(θ)>0 ∀ θ∈Θ. Missing values of X are imputed from their posterior predictive distribution implied by this model. One way to draw from this distribution is to use the following Gibbs sampler algorithm.^4^ First, replace the missing values by arbitrary starting values. A single iteration of the Gibbs sampler then consists of *K* steps, in the *k*th of which the values of $\left\{X_{\mathrm{ik}}:i\in M_{k}\right\}$ are updated. Let $X_{i,-k}^{*}=(X_{i1}^{*},\ldots ,X_{i,k-1}^{*},X_{i,k+1}^{*},\ldots ,X_{\mathrm{iK}}^{*}{)}^{⊤}$, where $X_{\mathrm{ij}}^{*}$ equals its observed value $X_{\mathrm{ij}}$ if *R*_*ij*_ = 1 and equals its most recently sampled value if *R*_*ij*_ = 0. Let $f_{k}(X_{k}|X_{-k},\theta )$ and $f_{-k}(X_{-k}|\theta )$ denote the conditional distributions of *X*_*k*_ given $X_{-k}$ and the marginal distribution of $X_{-k}$, respectively, implied by joint model f(X|θ). The *k*th step consists of first sampling θ from the distribution proportional to $p(\theta )\overset{n}{\underset{i=1}{\Pi }}f_{k}(X_{\mathrm{ik}}|X_{i,-k}^{*},\theta {)}^{R_{\mathrm{ik}}}f_{-k}(X_{i,-k}^{*}|\theta )$ and then, using this sampled value of θ, sampling *X*_*ik*_ from $f_{k}(X_{\mathrm{ik}}|X_{i,-k}^{*},\theta )$ for each $i\in M_{k}$. These *K* steps are iterated until the imputed variables converge in distribution.

In FCS MI, a set of *K* conditional models $\left\{g(X_{k}|X_{-k},\varphi_{k}):k=1,\ldots ,K\right\}$ is specified for the distribution of each *X*_*k*_ given the remaining variables. Also specified is a non-informative prior $p(\varphi_{k})$ (k=1,…,K) for the parameters $\varphi_{k}$ in each of these models. Let $\Phi_{k}$ denote the parameter space of $\varphi_{k}$ and assume that $p_{k}(\varphi_{k})>0$ ∀ $\varphi_{k}\in \Phi_{k}$. As with the Gibbs sampler, the missing values are first replaced by arbitrary starting values and a single iteration of the FCS algorithm consists of *K* steps. The *k*th step involves first sampling $\varphi_{k}$ from the distribution proportional to $p(\varphi_{k})\overset{n}{\underset{i=1}{\Pi }}g_{k}(X_{\mathrm{ik}}|X_{i,-k}^{*},\varphi_{k}{)}^{R_{\mathrm{ik}}}$ and then, using this sampled value of $\varphi_{k}$, sampling *X*_*ik*_ from $g_{k}(X_{\mathrm{ik}}|X_{i,-k}^{*},\varphi_{k})$ for each $i\in M_{k}$.

Hughes et al.^4^ noted that FCS MI and the Gibbs sampler algorithm (and hence joint model MI) are equivalent when, for each *k*, the parameters θ of the joint model can be partitioned (possibly after reparameterisation) into a set of parameters that describe only the conditional distribution of *X*_*k*_ given $X_{-k}$ and a set of parameters that describe only the marginal distribution of $X_{-k}$, and p(θ) implies that these two parameter sets are a priori independent. More formally, for each *k* (k=1,…,K), let $\varphi_{k}=\varphi_{k}(\theta )$ and $\varphi_{-k}=\varphi_{-k}(\theta )$ be functions of θ such that $f_{k}(X_{k}|X_{-k},\theta )=f_{k}(X_{k}|X_{-k},\varphi_{k})$ and $f_{-k}(X_{-k}|\theta )=f_{-k}(X_{-k}|\varphi_{-k})$. Then joint model MI is equivalent to FCS MI with conditional models $f_{k}(X_{k}|X_{-k},\varphi_{k})$ (k=1,…,K) if the prior distribution, $p(\varphi_{k},\varphi_{-k})$, of $\left(\varphi_{k},\varphi_{-k}\right)$ implied by p(θ) can be factorised as $p(\varphi_{k},\varphi_{-k})=p_{k}(\varphi_{k})p_{-k}(\varphi_{-k})$ for each *k*. This ability of the prior to be so factorised has been called the ‘non-informative margins condition’.^4^

The non-informative margins condition cannot hold unless $\varphi_{k}$ and $\varphi_{-k}$ are distinct parameters, i.e. unless their joint parameter space is the product of their individual parameter spaces. In Section 4.1, we look at two examples where θ cannot be partitioned into distinct parameters $\varphi_{k}$ for the conditional distribution and $\varphi_{-k}$ for the marginal distribution. When θ cannot be partitioned into distinct parameters, data on $X_{-k}$ indirectly provides information on $\varphi_{k}$ through the information it provides on $\varphi_{-k}$. This indirect information is used in the Gibbs sampler but not in FCS MI.

An important theoretical result about the asymptotic relation between FCS and joint model MI was provided by Liu et al.^5^ This result can apply even when the non-informative margins condition is not satisfied. They defined the set of conditional models $\left\{g(X_{k}|X_{-k},\varphi_{k}):k=1,\ldots ,K\right\}$ to be *compatible* with a joint model f(X|θ) if (i) for each θ∈Θ and for each k=1,…,K, there exists a value of $\varphi_{k}\in \Phi_{k}$ such that $g_{k}(X_{k}|X_{-k},\varphi_{k})=f_{k}(X_{k}|X_{-k},\theta )$, and (ii) for each k=1,…,K and for each value of $\varphi_{k}\in \Phi_{k}$, there exists at least one value of θ∈Θ such that $g_{k}(X_{k}|X_{-k},\varphi_{k})=f_{k}(X_{k}|X_{-k},\theta )$.

Theorem 1 of Liu et al.^5^ says that if (i) the set of conditional models is compatible with a joint model, (ii) this joint model is correctly specified and (iii) the data are missing at random (MAR), then the total variation distance between the distribution of the imputed data obtained from FCS MI and the distribution of the imputed data obtained from joint model MI tends to zero in probability as the sample size tends to infinity. More informally, we can say that the distribution of the imputed data is asymptotically the same whether one imputes by FCS MI or by joint model MI using the corresponding joint model. Liu et al.^5^ say that ‘iterative imputation [i.e. FCS MI] and joint Bayesian imputation [i.e. joint model MI] are asymptotically the same’ (p. 161).

Three comments are worth making. First, asymptotic equivalence of the imputation distributions of FCS MI and joint model MI does not mean that the two resulting Rubin's Rules estimators of β have the same asymptotic efficiency, as we illustrate in Section 4. Second, when the joint model is misspecified, FCS MI and joint model MI may use different imputation distributions for the missing data, even asymptotically, as we illustrate in Section 5. Third, suppose that X can be partitioned as $X=(Z^{⊤},X_{A}^{⊤}{)}^{⊤}$, where Z is fully observed. Then, conditional models for the elements of Z are not used in the FCS MI algorithm and need not be specified. Likewise, joint model MI requires only a model $f(X_{A}|Z,\theta )$ for the conditional distribution of $X_{A}$ given Z; the marginal distribution of Z is not used and no model for it need be specified. So, if the conditional models for $X_{A}$ are compatible with $f(X_{A}|Z,\theta )$, and if $f(X_{A}|Z,\theta )$ is correctly specified and the data are MAR, then FCS MI and joint model MI impute missing $X_{A}$ from the same distribution asymptotically.

## 3 The RGL model

Let Y and W be categorical and continuous variables, respectively. A categorical variable with *m* > 2 levels is coded as *m* − 1 indicator variables. The RGL model combines a log linear model with a conditional normal model:

(1) $$P(Y=y)=\frac{\text{exp}(\theta_{y}^{⊤}y+y^{⊤}\theta_{\mathrm{yy}}y)}{\sum_{y'}\text{exp}(\theta_{y}^{⊤}y'+y{'}^{⊤}\theta_{\mathrm{yy}}y')}$$

(2) $$W|Y∼N(\theta_{w0}+\theta_{\mathrm{wy}}Y,\theta_{v})$$

where $\theta_{y}$ and $\theta_{w0}$ are parameter vectors and $\theta_{\mathrm{yy}},\theta_{\mathrm{wy}}$ and $\theta_{v}$ are parameter matrices. Matrix $\theta_{\mathrm{yy}}$ is strictly upper triangular and $\theta_{v}$ is positive definite. Note that the term $\theta_{y}^{⊤}y+y^{⊤}\theta_{\mathrm{yy}}y$ in equation (1) means that the log linear model includes main effects for Y and all pairwise interactions between pairs of elements of Y. The *mix* library^7^ in R can be used to fit this model and to perform joint model MI based on it.

This RGL model implies that the conditional distribution of any element of W given Y and the remaining elements of W is normal with main effects only. It can also be shown that the RGL model implies that the conditional distribution of any categorical variable in Y given W and the remaining categorical variables has the form of a multinomial logistic regression with main effects only. If this categorical variable is binary, the multinomial logistic regression is just ordinary (binary) logistic regression. Expressions for the log odds ratios (LORs) in this logistic regression in terms of $\theta_{y},\theta_{\mathrm{yy}},\theta_{w0},\theta_{\mathrm{wy}}$ and $\theta_{v}$ are given in Appendix 1. Therefore, if these linear and logistic regressions are used as the conditional models in FCS MI, they are compatible with the RGL joint model. It follows from Theorem 1 of Liu et al.^5^ that if the RGL model is correctly specified and the data are MAR, then FCS MI and joint model MI asymptotically impute from the same distribution.

As mentioned at the end of Section 2, if some elements Z of Y and/or W are fully observed, conditional models are not required for them in FCS MI and they can be conditioned on in joint model MI. Using Y and W now to denote the categorical and continuous variables not included in Z, the resulting joint model is

(3) $$P(Y=y|Z=z)=\frac{\text{exp}(\theta_{y}^{⊤}y+y^{⊤}\theta_{\mathrm{yy}}y+y^{⊤}\theta_{\mathrm{yz}}z)}{\sum_{y'}\text{exp}(\theta_{y}^{⊤}y'+y{'}^{⊤}\theta_{\mathrm{yy}}y'+y{'}^{⊤}\theta_{\mathrm{yz}}z)}$$

(4) $$W|Y,Z∼N(\theta_{w0}+\theta_{\mathrm{wy}}Y+\theta_{\mathrm{wz}}Z,\theta_{v})$$

where $\theta_{\mathrm{yz}}\text{ and }\theta_{\mathrm{yy}}$ are strictly upper triangular. We call this the ‘RGL model conditional on Z' and write it as ‘CRGL(Z)’. This CRGL(Z) model imposes no constraints on the marginal distribution of Z. Like the RGL model, the CRGL(Z) model implies that the conditional distribution of any categorical variable in Y given W,Z and the remaining categorical variables has the form of a multinomial logistic regression. Expressions for the LORs in this logistic regression are given in Appendix 1. Again, if linear and logistic regressions with main effects only are used as the conditional models in FCS MI, they are compatible with the CRGL(Z) joint model. So, it follows that if the CRGL(Z) model is correctly specified and the data are MAR, then FCS MI and joint model MI using the CRGL(Z) model asymptotically impute from the same distribution. Moreover, since the RGL model implies the CRGL(Z) model, it follows that if the RGL model is correctly specified and the data are MAR, then FCS MI, joint model MI using the CRGL(Z) and joint model MI using the RGL model all asymptotically impute from the same distribution.

Note that, unlike the RGL model, the CRGL(Z) model cannot be fitted using the R *mix* library,^7^ unless Z includes only categorical variables.

Higher order interactions can be added to the log linear models of expressions (1) and (3). The conditional models of FCS MI then require additional interaction terms to remain compatible with this more general RGL or CRGL model. However, we focus on the log linear model with just main effects and pairwise interactions (expressions (1) and (3)) and study the impact of the absence of higher-order terms on the RE of FCS MI and joint model MI for inference about β.

## 4 Asymptotic RE of RGL versus FCS MI

### 4.1 Information in the marginal distribution

In Section 2, we noted that when the marginal distribution of $X_{-k}$ contains information about the parameters $\varphi_{k}$ of the conditional distribution of *X*_*k*_ given $X_{-k}$, joint model MI uses this information but FCS MI does not. In the RGL model, when *X*_*k*_ is an element of the vector of continuous variables W,θ can be partitioned into a priori independent parameters $\varphi_{k}$ and $\varphi_{-k}$. So, the marginal distribution of $X_{-k}$ provides no information about $\varphi_{k}$.^4^ However, when *X*_*k*_ is one of the categorical variables in Y, two assumptions of the RGL model make the marginal distribution of $X_{-k}$ informative.^4^

First, expressions (1) and (2) imply that the marginal distribution of W is a mixture of normal distributions. There is no way to parameterise this marginal distribution more parsimoniously than by using all of $\theta =(\theta_{y},\theta_{\mathrm{yy}},\theta_{w0},\theta_{\mathrm{wy}},\theta_{v})$. Therefore, $\varphi_{-k}=\theta$.

Second, suppose for simplicity that there are no continuous variables W, so that the RGL reduces to a log linear model, and that all the categorical variables *Y*_1_, …, *Y*_*L*_ are binary. The inclusion of only main effects and pairwise interactions in the log linear model of equation (1) means there are L(L+1)/2 parameters. The conditional probability that any one variable, say *Y*_1_, equals one given the others is the logistic regression form $P(Y_{1}=1|Y_{2},\ldots ,Y_{L})=\text{expit}(\theta_{1}+\sum_{j=2}^{L}\theta_{1j}Y_{j})$, where $\theta_{10},\theta_{12},\ldots ,\theta_{1L}$ are parameters. That leaves L(L-1)/2 parameters to describe the marginal distribution of $\left(Y_{2},\ldots ,Y_{L}\right)$. When $L\geq 4,L(L-1)/2<2^{L-1}-1$, the number of parameters needed for a saturated model for $\left(Y_{2},\ldots ,Y_{L}\right)$. This raises the possibility that the marginal distribution of $\left(Y_{2},\ldots ,Y_{L}\right)$ may depend on $\theta_{1},\theta_{12},\ldots ,\theta_{1L}$, and indeed this is so (see online Appendix). Thus, the marginal distribution contains information about the conditional distribution. This argument extends easily to the general case where L≥4, categorical variables have more than two categories, and/or there are continuous variables W.

In the remainder of this section, we study how much this information in the margins affects the asymptotic RE of the Rubin's Rules estimator of β using FCS MI compared to the estimator using joint model MI.

### 4.2 One binary and two continuous variables

Suppose that data are generated by the RGL model used by Hughes et al.^4^

$$\begin{array}{cc} Y & ∼\text{Bernoulli}(p)\\ W_{1}|Y & ∼\text{Normal}(10+\gamma_{1}Y,9)\\ W_{2}|Y,W_{1} & ∼\text{Normal}(9+8/9+W_{1}/9+\gamma_{2}Y,8+8/9) \end{array}$$

where *p* = 0.1 or 0.3, and *γ*_1_ and *γ*_2_ each equal 1, 2, 3 or 4, and *W*_1_ and *W*_2_ are fully observed (Hughes et al.^4^ considered only *p* = 0.3 and $\gamma_{1}=\gamma_{2}=1$ or $\gamma_{1}=\gamma_{2}=3$). This special case of the RGL model with only one binary variable is called the linear discriminant analysis (LDA) model.^10^ Using the formula in Appendix 1, it follows that $P(Y=1|W_{1},W_{2})=\text{expit}(\beta_{0}+\beta_{1}W_{1}+\beta_{2}W_{2})$, where $\beta =(\beta_{0},\beta_{1},\beta_{2}{)}^{⊤}=$ $\left(\text{logit}(0.3)-10\gamma_{1}/9-89\gamma_{2}/80-\gamma_{1}^{2}/18-9\gamma_{2}^{2}/160,\gamma_{1}/9-\gamma_{2}/80,9\gamma_{2}/80\right)$. Since the standard deviations of *W*_1_ and *W*_2_ are both approximately 3, the LORs *β*_1_ and *β*_2_ are very large when *γ*_1_ and/or *γ*_2_ equals 3 or 4 (e.g. 0.39 and 0.45 when $\gamma_{1}=\gamma_{2}=4$). Nevertheless, we include these scenarios in order to investigate what happens in situations of strong associations. More likely scenarios are $\gamma_{1}=\gamma_{2}=1$ and $\gamma_{1}=\gamma_{2}=2$; $\left(\beta_{1},\beta_{2}\right)$ is then either (0.099, 0.113) or (0.197, 0.225).

If *Y* is fully observed, β can be estimated by logistic regression or by LDA. The former makes no assumption about the marginal distribution of $(W_{1},W_{2}{)}^{⊤}$, whereas LDA assumes that it is a mixture of two normal distributions. LDA is known to be more efficient (in finite samples and asymptotically) than logistic regression when the LDA model is correctly specified, especially when *β*_1_ and *β*_2_ are large or when *p* is close to 0 or 1.^10,11^

When *Y* is partially observed, β can be estimated by using RGL MI or FCS MI and then analysing the imputed data using logistic regression or LDA. Like LDA, RGL MI assumes normality of $(W_{1},W_{2}{)}^{⊤}$ given *Y*. Like logistic regression, FCS MI does not assume this. Therefore, if RGL MI and logistic regression analysis are used, the imputer is assuming more than the analyst.^12^ If FCS MI and LDA are used, the analyst is assuming more than the imputer. Otherwise, analyst and imputer are making the same assumptions.

For fully observed *Y*, Table 1 shows the asymptotic RE of LDA compared to logistic regression when $\left(\gamma_{1},\gamma_{2})=(1,1\right)$, (2, 2) or (3, 3). This was calculated using Monte Carlo integration to evaluate expected information matrices. Results for other $\left(\gamma_{1},\gamma_{2}\right)$ values are shown in Table 4 of the online Appendix. It is seen that LDA can be more efficient than logistic regression, but that the difference is small unless *γ*_1_ and *γ*_2_ are large and is greater when *p* = 0.1 than when *p* = 0.3. The largest asymptotic RE when $\gamma_{1}\leq 2$ and $\gamma_{2}\leq 2$ was 104%, although it did rise to 142% when $\gamma_{1}=\gamma_{2}=4$ and *p* = 0.1.

**Table 1.** Percentage asymptotic REs of LDA versus logistic regression analysis when using complete data.

			Regression coefficient		
*p*	*γ*_1_	*γ*_2_	Intercept	*W*_1_	*W*_2_
0.1	1	1	99.4	100.4	100.3
0.1	2	2	103.7	103.3	103.6
0.1	3	3	116.6	112.7	114.0
0.3	1	1	99.2	100.2	100.1
0.3	2	2	101.7	101.7	101.8
0.3	3	3	109.5	107.0	107.6

These complete-data results suggest RGL MI may often not be much more efficient than FCS MI when *Y* is partially observed. To investigate this, we assumed that *Y* is missing with probability 0.5, either completely at random or at random with probability $P(R=0|W_{1},W_{2})=\text{expit}(c-W_{1}/3)$ (*c* was chosen to give P(R=1)=0.5). Using the formula in Theorem 1 of Robins and Wang,^13^ we calculated the asymptotic REs of RGL MI versus FCS MI for the Rubin's Rules estimators. This is the RE for an infinite sample size and M=∞ imputations. Monte Carlo integration was used to evaluate the expectations in the Robins and Wang formula. We considered four analyses: logistic regression, LDA, linear regression of *W*_2_ on *Y* and *W*_1_, and estimation of the marginal mean of *Y*.

Table 2 shows results for $\left(\gamma_{1},\gamma_{2})=(1,1\right)$, (2, 2) and (3, 3). Results for other $\left(\gamma_{1},\gamma_{2}\right)$ values are in Table 5 in the online Appendix. As expected, RGL MI is only slightly more efficient than FCS MI unless *γ*_1_ and/or *γ*_2_ are large, and the efficiency gain is greater for *p* = 0.1 than for *p* = 0.3. Efficiency gains are much greater for the logistic regression analysis than for the other three analyses but are still ≤10% unless $\gamma_{1}>2$ or $\gamma_{2}>2$.

**Table 2.** Percentage asymptotic REs of RGL MI versus FCS MI for four different analysis models.

		Analysis and regression coefficient									
*γ*_1_	*γ*_2_	E(Y)	lr(0)	lr(*W*_1_)	lr(*W*_2_)	ld(0)	ld(*W*_1_)	ld(*W*_2_)	ln(0)	ln(Y)	ln(*W*_1_)
*p* = 0.1 and MCAR											
1	1	99.9	100.4	100.3	100.4	100.0	100.1	100.1	100.0	100.1	100.0
2	2	99.9	106.6	104.5	105.1	101.8	101.3	101.5	100.1	101.5	100.3
3	3	100.1	123.9	117.3	119.1	105.8	104.5	105.0	100.9	104.5	101.5
*p* = 0.1 and MAR											
1	1	99.8	100.5	100.1	100.3	100.0	99.8	100.0	100.0	100.0	100.0
2	2	102.2	110.1	108.4	106.3	103.7	103.7	102.1	100.5	102.0	101.0
3	3	107.6	138.2	134.1	125.4	111.8	113.6	108.1	103.8	107.2	105.8
*p* = 0.3 and MCAR											
1	1	100.0	100.2	100.2	100.1	100.0	100.1	100.0	100.0	100.0	100.0
2	2	100.1	103.7	102.4	102.6	101.1	100.8	100.7	100.1	100.7	100.2
3	3	100.6	114.6	109.9	110.8	104.0	102.9	103.0	100.9	102.7	101.3
*p* = 0.3 and MAR											
1	1	100.0	100.2	100.2	100.1	100.0	100.0	99.9	100.0	99.9	100.0
2	2	100.6	104.2	103.2	103.0	101.4	101.3	101.0	100.2	100.9	100.4
3	3	103.2	117.6	114.5	113.3	105.8	106.1	104.5	101.7	103.9	102.9

*E*(*Y*): marginal mean of *Y*; lr: logistic regression; ld: linear discriminant analysis; ln: linear regression; (*V*): the regression coefficient associated with variable *V* (with (0) meaning the intercept); MAR: missing at random; MCAR: missing completely at random.

It is interesting that FCS MI is less efficient than RGL MI when the analysis is LDA. This shows that, for the RGL model, asymptotic efficiency is lost by the imputer assuming less than the analyst. Meng^12^ gave a different example of an imputer assuming less than an analyst and showed there was no loss of asymptotic efficiency in that case. It is also interesting that RGL MI is more efficient than FCS MI when the analysis is logistic regression. This is an example of the imputer assuming more than the analyst. Since FCS MI with an infinite number of imputations followed by logistic regression analysis is asymptotically equivalent to logistic regression analysis using only complete cases (because individuals with missing outcome provide no information in logistic regression when all covariates are fully observed), the greater efficiency of RGL MI followed by logistic regression analysis is an illustration of ‘super-efficiency’.^12,14^

Next, we investigated whether the asymptotic REs in Table 1 reflect the REs in finite samples. Table 6 in the online Appendix shows, for two scenarios, the RE of LDA using complete data versus logistic regression using complete data for a variety of sample sizes. The REs were estimated using 10,000 simulated datasets. Table 7 in the online Appendix shows, for the same two scenarios (and using the same 10,000 simulated datasets), the finite-sample RE of RGL MI versus FCS MI (using *M* = 50 imputations) for the four analyses of Table 2. The finite-sample REs are similar to the asymptotic REs. Note that, as expected, the Rubin's Rules point estimators were approximately unbiased for all methods (data not shown).

### 4.3 Four binary variables

Now suppose that data are generated by the log linear model with *L* = 4 binary variables, *P*(*Y*_1_, *Y*_2_, *Y*_3_.*Y*_4_) ∝ exp$({\sum^{4}}_{j=1}\theta_{j}Y_{j}+{\sum^{3}}_{j=1}{\sum^{4}}_{k=2}\theta_{\mathrm{jk}}Y_{j}Y_{k})$, where *θ_j_* = −0.5 for *j* = 2, 3, 4; *θ_jk_* = 0.5 for (*j*, *k*) = (2, 3), (2, 4), (3, 4); (*θ*_12_, *θ*_13_, *θ*_14_) = (0.33, 0.67, 1.00), (0.67, 1.33, 2.00), (1, 2, 3) or (3, 3, 3); and *θ*_1_ is chosen to make *P*(*Y*_1_ = 1) = 0.3. The parameters ***β*** = (*θ*_1_, *θ*_12_, *θ*_13_, *θ*_14_)^⊤^ can be estimated by fitting either this log linear model or the logistic regression model *P*(*Y*_1_ = 1 | *Y*_2_, *Y*_3_, *Y*_4_) = expit$(\theta_{1}+{\sum^{4}}_{j=2}\theta_{1j}Y_{j})$ that it implies. We calculated the RE of these two methods when the data were complete. We also calculated the RE of RGL MI versus FCS MI when *Y*_1_ was missing with probability 0.5 completely at random or at random and analysis was either by logistic regression or by fitting the log linear model.

Detailed results are given in the online Appendix. In summary, we found that analysing the complete data by fitting the log linear model was hardly any more efficient than using logistic regression: all REs were less than 107%. Likewise, analysing RGL MI was not much more efficient than FCS MI: all REs were less than 116% when analysis was by logistic regression and all were less than 108% when analysis was by fitting the log linear model. These maximum REs required strong associations between variables, i.e. $\left(\theta_{12},\theta_{13},\theta_{14})=(3,3,3\right)$. With more moderate associations, REs did not exceed 105%. It appears therefore that the marginal distribution of $\left(Y_{2},Y_{3},Y_{4}\right)$ contains little information about *θ*_1_, *θ*_12_, *θ*_13_ and *θ*_14_. We also considered data-generating mechanisms with *L* = 6 variables or changed the parameters of interest to $\beta =(\theta_{4},\theta_{14},\theta_{24},\theta_{34}{)}^{⊤}$, i.e. parameters of the regression of *Y*_4_ on *Y*_1_, *Y*_2_ and *Y*_3_, so that the partially observed variable (*Y*_1_) was a covariate. In all cases, REs were less than 108% (data not shown).

### 4.4 Simulation study based on BCGS

To investigate RE of RGL MI versus FCS MI in a realistic setting, we carried out a simulation study based on real data from the BCGS. The BCGS is a follow-up study of a dietary intervention randomised controlled trial of pregnant women and their offspring.^8^ Participants in the original trial were followed up until offspring were five years old. When aged 25, these offspring were invited to participate in a follow-up study. There were 951 offspring in the trial, of whom 712 participated in the follow-up study.

For the simulation study, we considered eight variables: sex, childhood weight (at age 5), adult overweight (a binary indicator of BMI ≥25), ex-smoker and height (all at age 25), father's and mother's weights and father's social class. We considered as our analysis of interest a logistic regression of adult overweight on the other variables. Adult overweight and adult height were missing on (the same) 272 of the 951 offspring; ex-smoker and father's weight were missing on, respectively, 241 and 149 offspring. Sex was fully observed, and there were a total of 12 missing values on the remaining three variables. Among the 679 offspring with observed outcome, there were only 109 missing values, 98 of which were for father's weight.

Simulated datasets were created as follows. First, we fitted the RGL model of equations (1) and (2) to the BCGS data. Then, the fitted model was used to generate complete data on the eight variables for each of 951 hypothetical individuals independently. Missingness was then imposed using missingness models whose parameters were estimated from the BCGS data.

The simulation study was in two parts. In Part I, three of the continuous variables (father's, mother's and childhood weights) were treated as auxiliary variables, i.e. they were included in the imputation model but not in the analysis model; the other variables were included in both models. Using auxiliary variables in the imputation model may increase efficiency and make the MAR assumption more plausible.^15^ With auxiliary variables, it may be worth imputing a missing outcome even if the covariates in the analysis model are fully observed.

In Part II, there were no auxiliary variables: all eight variables were included in the analysis model. In the absence of auxiliary variables, Von Hippel^15^ recommended including all individuals in the imputation step but then excluding those with imputed outcomes before fitting the analysis model to the imputed data, in order to reduce bias caused by a possibly misspecified imputation model. This approach is valid when (i) the data are MAR, (ii) the model for the conditional distribution of outcome given covariates implied by the imputation model is the same as the analysis model and (iii) the analysis model is correctly specified. We therefore analysed imputed data both before and after excluding imputed outcomes. As the proportion of missing covariate values among offspring with observed outcome was small in the BCGS dataset, we increased this proportion for the simulation (Part II only).

For both parts, we checked that the RGL model was not an obvious poor fit to the BCGS data by comparing the LORs from a complete-case logistic regression of adult overweight on the other variables with the corresponding LORs implied by the fitted RGL model. The estimates were similar, providing some reassurance.

We considered several simulation scenarios, by varying the strength of association between the auxiliary variables and outcome (in Part I) and the amount of missingness in the covariates (in Part II). For each scenario, we simulated 1000 datasets using R. RGL MI was performed using the *mix* library in R; FCS MI used the *ice* package in STATA; *M* = 100 imputations were used. Full details are given in the online Appendix.

To summarise the results, in Part I the maximum RE of RGL MI versus FCS MI was 104% (this was for LOR of ex-smoker). In Part II, the covariate with the highest RE was ex-smoker; this RE was 105% when 33% of ex-smoker values were missing and 111% when 54% of ex-smoker values were missing. When imputed outcomes were excluded before fitting the analysis model, these maximum REs decreased to, respectively, 102% and 108%. Full results are in the online Appendix.

## 5 Robustness of FCS and joint model MI

In Section 4, we demonstrated that RGL MI can be more efficient than FCS MI but that the gains seem to be small unless associations between variables are very strong. In this section, we show that these efficiency gains can come at the price of bias when the RGL model is misspecified. In Section 5.1, we modify the RGL model used in Section 4.2 so that *W*_1_ is not normally distributed given *Y*. It is now a CRGL(*W*_1_) model but not a RGL model. We show that when *W*_1_ is fully observed and *Y* is partially observed, logistic regression gives unbiased estimation when imputation is by FCS MI but not when RGL MI is used. In Section 5.2, we modify the log linear model of Section 4.3 by introducing a third-order interaction between *Y*_2_, *Y*_3_ and *Y*_4_. The log linear model with only main effects and pairwise interactions is now misspecified. We show that this causes bias when RGL MI is used but not when FCS MI is used, because FCS MI makes no assumption about the distribution of fully observed variables. In the online Appendix, we present a realistic analysis of data from the NCDS,^9^ which illustrates that use of RGL MI can lead to serious bias in a situation where FCS MI does not.

### 5.1 One binary and two continuous variables

We simulated data from the following modification of the RGL model of Section 4.2.

(5) $$W_{1}∼\text{Gamma}(2,2)$$

(6) $$Y|W_{1}∼\text{Bernoulli}(\text{expit}(-1.9+W_{1}))$$

(7) $$W_{2}|Y,W_{1}∼\text{Normal}(10+\gamma Y+W_{1},9)$$

Now, *W*_1_ is no longer normally distributed given *Y*. This is a CRGL(*W*_1_) model but not a RGL model. We considered eight scenarios defined by the value of *γ* (1 or 3), by which variables were partially observed (either just *Y* or both *Y* and *W*_2_), and by whether data were missing completely at random (MCAR) or MAR. The probability that each partially observed variable was observed was 0.5 if data were MCAR and $\text{expit}(-1+W_{1})$ if MAR (this gives a marginal probability of missingness of 0.5). In scenarios where both *Y* and *W*_2_ were missing, their missingness was independent given *W*_1_. For each scenario, we generated 1000 datasets each of size *n* = 1000.

Missing data were imputed using either FCS MI or RGL MI, with *M* = 50 imputations. Four analyses were carried out: estimating *E*(*Y*) by the sample mean of *Y*; estimating parameters *β*_0_, *β*_1_ and *β*_2_ of $P(Y=1|W_{1},W_{2})=\text{expit}(\beta_{0}+\beta_{1}W_{1}+\beta_{2}W_{2})$ using either logistic regression or LDA; and estimating the parameters of the linear regression of *W*_2_ on *Y* and *W*_1_. Using the formula in Appendix 1, $\left(\beta_{0},\beta_{1},\beta_{2})=(-3.067,0.889,0.111\right)$ when *γ* = 1 and (-5.733,0.667,0.333) when *γ* = 3.

Since *W*_1_ is fully observed and the CRGL(*W*_1_) model is correctly specified, logistic regression analysis of data imputed by FCS MI should yield asymptotically unbiased estimators, whereas imputing using RGL MI or analysing using LDA may yield asymptotically biased estimators, because the RGL model is misspecified. Table 3 shows the means of the parameter estimates when *γ* = 1. Results for *γ* = 3 are given in Table 8 of the online Appendix. It can be seen that LDA gives a biased estimate of the LOR of *W*_1_ whether one uses complete data, FCS MI or RGL MI. Provided that the analysis is logistic regression or linear regression, there is no bias when using complete data or FCS MI. For RGL MI, on the other hand, there is bias in the coefficient of *W*_1_ in the logistic regression and linear regression analyses. These biases are small for linear regression and are slightly greater for *γ* = 3 than for *γ* = 1.

**Table 3.** Mean estimates when RGL model is misspecified and *γ* = 1.

	Analysis and regression coefficient									
	E(Y)	lr(0)	lr(*W*_1_)	lr(*W*_2_)	ld(0)	ld(*W*_1_)	ld(*W*_2_)	ln(0)	ln(Y)	ln(*W*_1_)
True	0.300	−3.067	0.889	0.111	−3.067	0.889	0.111	10.000	1.000	1.000
cdata	0.303	−3.091	0.890	0.113	−3.232	0.987	0.113	10.001	1.012	0.995
*Y* MCAR, and *W*_1_ and *W*_2_ fully observed										
FCS	0.304	−3.101	0.895	0.114	−3.241	0.992	0.114	9.999	1.011	0.996
RGL	0.296	−3.276	1.009	0.114	−3.456	1.131	0.114	10.024	1.013	0.979
Both *Y* and *W*_2_ MCAR, and *W*_1_ fully observed										
FCS	0.304	−3.105	0.900	0.113	−3.245	0.997	0.113	10.006	0.998	0.993
RGL	0.296	−3.287	1.014	0.114	−3.468	1.136	0.114	10.032	1.004	0.974
All of *Y, W*_1_ and *W*_2_ MCAR										
FCS	0.304	−3.124	0.916	0.113	−3.255	1.004	0.113	10.003	0.996	0.996
RGL	0.296	−3.299	1.028	0.114	−3.466	1.140	0.114	10.028	1.001	0.978
*Y* MAR, and *W*_1_ and *W*_2_ fully observed										
FCS	0.303	−3.102	0.891	0.114	−3.237	0.983	0.114	9.998	1.012	0.998
RGL	0.303	−3.271	1.047	0.114	−3.445	1.161	0.114	10.024	1.012	0.972
Both *Y* and *W*_2_ MAR, and *W*_1_ fully observed										
FCS	0.304	−3.111	0.898	0.114	−3.247	0.989	0.114	10.005	1.004	0.993
RGL	0.303	−3.290	1.051	0.115	−3.465	1.166	0.115	10.027	1.010	0.971

Lr: logistic regression; ld: linear discriminant analysis; ln: linear regression; (*V*): the regression coefficient associated with variable *V* (with (0) meaning the intercept); MCAR: missing completely at random; FCS: full-conditional specification; RGL: restricted general location; MAR: missing at random.

For the scenarios where *Y* and *W*_2_ were both partially observed, we also applied logistic regression to the datasets imputed by RGL MI after excluding the individuals whose *Y* value had been imputed. Similarly, we applied linear regression to the imputed datasets after excluding the individuals whose *W*_2_ value had been imputed. This strategy of excluding imputed outcomes before analysing the data has been advocated by Von Hippel^15^ as a way of reducing bias caused by a possibly misspecified imputation model. Table 9 in the online Appendix shows the results. Most or all of the bias has been removed for logistic regression but none has been removed for linear regression. Note that Von Hippel did not recommend this approach when MI is done using strong auxiliary variables.

Finally, in scenarios where *Y*_1_ and *W*_2_ were both MCAR, we additionally imposed 10% missingness on *W*_1_. Tables 3 and 8 show that although there is some bias for logistic regression analysis when FCS MI is used, this is much less than when RGL MI is used.

### 5.2 Four binary variables

Consider again the data generating mechanism of Section 4.3 but now suppose that the log linear model contains an additional third-order interaction:

$$\begin{array}{c} \text{log}P(Y_{1},Y_{2},Y_{3},Y_{4})\propto \text{exp}(\sum_{j=1}^{4}\theta_{j}Y_{j}+\sum_{j=1}^{3}\sum_{k=2}^{4}\theta_{\mathrm{jk}}Y_{j}Y_{k}-2Y_{2}Y_{3}Y_{4}) \end{array}$$

The log linear model with only main effects and pairwise interactions is now misspecified. Table 10 in the online Appendix shows, for four true values of $\beta =(\theta_{12},\theta_{13},\theta_{14}{)}^{⊤}$, the mean estimates of β when the complete data or imputed data are analysed by logistic regression or by fitting the log linear model. This shows that there is bias when fitting the log linear model (even to the complete data) or when imputation is by RGL MI, and that there is no bias when the complete data or data imputed by FCS MI are analysed by logistic regression. Note that, unlike the normality assumption of the RGL model, which is an intrinsic feature of that model, higher-order interactions can be allowed in the RGL model, but in practice, this might not be done.

## 6 Discussion

FCS and joint model MI yield imputed data with the same asymptotic distribution when the conditional models used by FCS MI are compatible with the joint model. However, we have shown that this asymptotic equivalence in terms of the imputation distribution does not imply that FCS and joint model MI yield equally asymptotically efficient estimates of the parameters in the analysis model. Moreover, FCS MI can be more robust than joint model MI to misspecification of the joint model. We focussed on the RGL model. The efficiency gain from using joint model MI with this model (RGL MI) rather than the corresponding FCS MI appears to be small, except when the outcome is categorical and has a large proportion of missingness and very strong associations exist between the outcome and covariates. On the other hand, we have shown that if the RGL model is misspecified, RGL MI can be much more biased than FCS MI in this same situation, even when covariate-outcome associations are weaker.

Robustness of RGL MI can be improved by including additional interactions in the model (this could have been done in, e.g. the analysis of the NCDS data in the online Appendix) or by conditioning on fully observed variables Z (the CRGL(Z) model). However, the R *mix* library cannot be used to fit the CRGL(Z) model or to carry out joint model MI with this model, unless Z includes only categorical variables. Bayesian modelling software, such as WinBUGS, could be used, but this requires more specialist programming skills. Robustness of RGL MI can also be improved by excluding individuals with imputed outcomes from the analysis. This approach was advocated by Von Hippel (2007),^15^ at least when there are no strong auxiliary variables. In the absence of auxiliary variables and when data are MAR, excluding individuals with imputed outcomes causes no loss of efficiency when the analysis is by linear regression or LDA, and causes no bias and is likely to cause little loss of efficiency when analysis is by logistic regression (especially when those with missing outcomes also have missing values in covariates). Conversely, Sullivan et al.^16^ show that excluding imputed outcomes can cause significant bias when auxiliary variables are strongly associated with both the outcome and missingness in that outcome. They did not, however, investigate situations where the imputation model is misspecified.

Although careful assessment of goodness of fit of an imputation model could detect poor fit of that model, we suspect that this may often not be done in practice. For this reason, FCS MI may be safer than RGL MI when a large proportion of outcomes are missing, unless imputed outcomes are excluded from the subsequent analysis. Since, as Sullivan et al.^16^ noted, this exclusion can itself induce another bias, we suggest that a good approach may be to use FCS MI imputing the outcome last and including imputed outcomes in the analysis. Our results indicate that the efficiency loss from using FCS rather than joint model MI is unlikely to be significant in practice.

Several comparisons of joint model MI with FCS MI have been published (e.g. Lee and Carlin^17^ and Kropko et al.^18^ and references therein). However, these have focussed on joint model MI using a multivariate normal model. The distributions of the imputed data from this joint normal model MI and from FCS MI are not asymptotically equivalent, unless all variables are continuous. These published comparisons have generally noted little difference in efficiency, and relative robustness depended on how categorical variables were handled by joint normal model MI.

An alternative to RGL (or CRGL) MI and FCS MI is joint model MI under the latent normal model of Goldstein and Carpenter.^2,19^ This can be implemented using REALCOM-IMPUTE or the jomo package in R. This software allows conditioning on fully observed variables. This approach also extends to multi-level data by using random effects. Unlike the RGL model, the latent normal model does not imply conditional distributions that are linear or logistic/multinomial regressions. This is why we compared FCS MI with RGL MI rather than with joint model MI under the latent normal model. It also means that, in general, there is incompatibility (sometimes known as ‘uncongeniality’^12^) between a latent normal imputation model and a linear or logistic regression analysis model. Nevertheless, while some forms of incompatibility (uncongeniality) between imputation and analysis models (e.g. an imputation model that ignores an interaction present in the analysis model^12^) may cause substantial bias in the estimates of the parameters of the analysis model, other forms may often not matter in practice.^7^ Moreover, MI under the latent normal model may be more robust than RGL MI to model misspecification; more research on this is needed.

A limitation of our work is that, because it was not feasible to study every possible data-generating mechanism, we cannot rule out the possibility that there are scenarios in which large efficiency gains are possible without requiring strong associations between variables, although this seems unlikely. We focussed on parameter estimation. It is plausible that many of our conclusions could apply when the model of interest is used for prediction, since in that case the linear predictor is a weighted average of the individual parameters. However, further research is warranted into the RE and relative robustness of joint model MI and FCS MI when the ultimate aim is prediction, classification or clustering. Another direction of future research would be to compare FCS and RGL MI when data are missing not at random, the CRGL model is misspecified or FCS MI does not use compatible conditional models.^20^ It is possible that FCS MI using incompatible conditional models may be more efficient than joint model MI using a misspecified joint model, especially when those conditional models have been chosen to fit well to the observed data.

In conclusion, FCS MI may be preferable to joint model MI using the compatible joint model, viz. the RGL model: it is more robust and is usually only slightly less efficient.

## Appendix 1

### LORs implied by CGRL model

Consider the CRGL(Z) model of expressions (3) and (4). Partition Y as $Y=(Y_{A},Y_{B}^{⊤}{)}^{⊤}$, where *Y*_*A*_ is a binary variable (and not a dummy variable for a categorical variable with more than two levels). Similarly, partition $\theta_{y}$ as $\theta_{y}=(\theta_{\mathrm{yA}},\theta_{\mathrm{yB}}^{⊤}{)}^{⊤}$ and similarly with $\theta_{\mathrm{yy}},\theta_{\mathrm{yz}},\theta_{w0},\theta_{\mathrm{wy}}$ and $\theta_{\mathrm{wz}}$.

Equation (3) implies that

$$P(Y_{A}=1|Y_{B},Z)=\frac{\text{exp}(\theta_{\mathrm{yA}}+\theta_{\mathrm{yAyB}}^{⊤}Y_{B}+\theta_{\mathrm{yAz}}^{⊤}Z)}{1+\text{exp}(\theta_{\mathrm{yA}}+\theta_{\mathrm{yAyB}}^{⊤}Y_{B}+\theta_{\mathrm{yAz}}^{⊤}Z)}$$

By using Bayes' Theorem, it is easy to show that

$$P(Y_{A}=1|Y_{B},Z,W)=\frac{\text{exp}(\beta_{0}+\beta_{1}^{⊤}Y_{B}+\beta_{2}^{⊤}Z+\beta_{3}^{⊤}W)}{1+\text{exp}(\beta_{0}+\beta_{1}^{⊤}Y_{B}+\beta_{2}^{⊤}Z+\beta_{3}^{⊤}W)}$$

where

$$\begin{array}{c} \beta_{3}=\theta_{v}^{-1}\theta_{\mathrm{wyA}}\\ \beta_{0}=\theta_{\mathrm{yA}}-\theta_{w0}^{⊤}\beta_{3}-0.5\theta_{\mathrm{wyA}}^{⊤}\beta_{3}\\ \beta_{1}=\theta_{\mathrm{yAyB}}-\theta_{\mathrm{wyB}}^{⊤}\beta_{3}\\ \beta_{2}=\theta_{\mathrm{yAz}}-\theta_{\mathrm{wz}}^{⊤}\beta_{3} \end{array}$$

The CRGL(Z) model reduces to the RGL model when Z is empty. Hence, the above expressions for *β*_0_, $\beta_{1}$ and $\beta_{3}$ apply to the RGL model if $\beta_{2},\theta_{\mathrm{yAz}}$ and $\theta_{\mathrm{wz}}$ are omitted.
